# Democrats and Republicans Can Be Differentiated from Their Faces

**DOI:** 10.1371/journal.pone.0008733

**Published:** 2010-01-18

**Authors:** Nicholas O. Rule, Nalini Ambady

**Affiliations:** Department of Psychology, Tufts University, Medford, Massachusetts, United States of America; Harvard University, United States of America

## Abstract

**Background:**

Individuals' faces communicate a great deal of information about them. Although some of this information tends to be perceptually obvious (such as race and sex), much of it is perceptually ambiguous, without clear or obvious visual cues.

**Methodology/Principal Findings:**

Here we found that individuals' political affiliations could be accurately discerned from their faces. In Study 1, perceivers were able to accurately distinguish whether U.S. Senate candidates were either Democrats or Republicans based on photos of their faces. Study 2 showed that these effects extended to Democrat and Republican college students, based on their senior yearbook photos. Study 3 then showed that these judgments were related to differences in perceived traits among the Democrat and Republican faces. Republicans were perceived as more powerful than Democrats. Moreover, as individual targets were perceived to be more powerful, they were more likely to be perceived as Republicans by others. Similarly, as individual targets were perceived to be warmer, they were more likely to be perceived as Democrats.

**Conclusions/Significance:**

These data suggest that perceivers' beliefs about who is a Democrat and Republican may be based on perceptions of traits stereotypically associated with the two political parties and that, indeed, the guidance of these stereotypes may lead to categorizations of others' political affiliations at rates significantly more accurate than chance guessing.

## Introduction

People ubiquitously draw conclusions about others based on their appearance and behaviors [1], [2]. Although many aspects of our nonverbal behaviors and appearance may communicate information about us, perhaps the most important communicator of information about our traits, dispositions, and identities is the face [2]. People are known to form impressions of others from their faces instantaneously and automatically [3]. Moreover, these perceptions can have highly consequential outcomes, such as affecting the jobs that individuals are offered [4] their outcomes in court [5], and their financial success [6]–[8].

Some characteristics are known to be more legible from our faces than others. For instance, visually obvious characteristics such as age, race, and sex are rapidly and readily perceived from facial appearance [3], [9]–[10]. Yet there is also evidence that aspects of individuals that are considerably less obvious are also perceived somewhat effortlessly. For example, sexual orientation is perceived accurately, rapidly, and automatically from the face and its features [11]–[13]. The rates of accuracy in perceiving sexual orientation are not as high as those for age, race, and sex, however. Rather, characteristics such as sexual orientation and religious group membership tend to be fairly ambiguous to perceivers. Despite the perceptual ambiguity of these categories, perceivers' rates of accuracy in categorizing others along the dimensions of religion and sexual orientation are significantly greater than what would be expected from mere chance guessing [14]–[16]. Thus, even subtle differences in perceptual cues may lead to accurate perceptions.

One particularly consequential judgment is political candidates' actual electoral success based on perceivers' naïve judgments of personality traits from the candidates' faces. Several studies have found that judgments of competence and power from the faces of political candidates in Western cultures are significantly related to the candidates' margin of victory [17]–[19]. Indeed, even children's judgments of politicians' faces can predict their electoral success [20] and judgments of power and warmth from faces can predict electoral outcomes across cultures [19].

Given the ability of perceivers to infer the electoral success of political candidates from their faces and the importance and consequentiality of accurately perceiving others' group memberships, more generally, we wondered whether perceivers would be able to accurately categorize individuals according to their political group membership. Political party affiliation is an important and salient identity for many individuals. Membership in one political group versus another can imply an endorsement of various philosophies and ideals and the platforms of alternate political parties may therefore appeal to individuals possessing distinct traits or dispositions [21]. Considering the elective nature of choosing a political affiliation, we expected that political affiliation may represent an instance of a perceptually ambiguous category. If political affiliation was to be perceptible from facial appearance, we suspected that the rate of accuracy may be relatively low (albeit necessarily greater than chance guessing) and related to individuals' or communities' stereotypes about membership in a given political party—especially in the U.S., where the political community is largely dichotomously divided between Democrats and Republicans. To test these questions, we asked undergraduate participants to categorize as Democrats and Republicans the faces of professional politicians (Study 1) and their undergraduate peers (Study 2). We then related these categorizations to perceptions of traits from the targets' faces as a means of elucidating a possible mechanism for these effects (Study 3).

## Method

The current work was approved by the Institutional Review Board at Tufts University.

### Study 1

Can political affiliation be ascertained from an individual's face? To test this question, we asked participants to categorize the faces of professional politicians: the Democrat and Republican candidates from the 2004 and 2006 U.S. Senate elections.

Photos of the Democrat and Republican candidates from the 2004 and 2006 Senate elections were downloaded from the website of the Cable News Network (CNN; http://www.cnn.com/ELECTION/) or from the candidates' campaign websites. Each photo was cropped to the extremes of the targets' heads (top of head, bottom of chin, sides of hair or ears), converted to grayscale, and standardized for size. To avoid race-based stereotypes, racial minority candidates were excluded from the study. In total, there were 118 candidates: 59 Democrats (*n* = 15 women) and 59 Republicans (*n* = 5 women).

Twenty-nine undergraduate participants (*n* = 12 women) were instructed that they would be seeing a series of faces presented on a computer screen and that their task was to categorize each person as either a Democrat or Republican, using the “D” and “R” keys, respectively. Each image was presented in a random order and responses were collected using DirectRT software. After categorizing the 118 faces, participants were asked to volunteer their own political party membership (*n* = 23 Democrats, *n* = 6 Republicans) and to indicate whether they had recognized any of the targets that they categorized (*n* = 10 participants).

### Study 2

Study 1 examined whether career politicians' faces express information about their political affiliations. Study 2 extended this investigation to targets whose political group membership is less salient. We therefore asked perceivers to categorize the faces of college students belonging to Democrat and Republican clubs on campus from the senior portraits published in the targets' university yearbook.

Photos of self-identified Democrat (*n* = 30; *n* = 15 women) and Republican (*n* = 30; *n* = 9 women) undergraduates were digitally scanned from the senior yearbooks spanning years 2000–2008 of a private northeastern U.S. university. All photos were of the targets' senior portraits and targets had indicated their membership in either the university's Democrat or Republican student group, which was recorded in the yearbook. The photos were prepared using the same image standardization procedures as in Study 1. All of the targets were Caucasian, similarly dressed, and homogeneous for educational background and age. Independent coders rated each target's affect (*n* = 3; Cronbach's α = .80) or attractiveness (*n* = 12; Cronbach's α = .90) along a 7-point scale anchored at either (1) *Neutral* or *Not at all attractive* and (7) *Happy* or *Very attractive*, respectively. The Democrat and Republican targets differed on neither dimension: *t*_Affect_(58) = 0.77, *p* = .44; *t*_Attract_(58) = 0.09, *p* = .93.

Twenty-four undergraduates (*n* = 14) at a different university categorized each face following the same procedures as in Study 1. No participant recognized any of the targets but a computer malfunction caused participants' political affiliations to go unrecorded.

### Study 3

Studies 1 and 2 tested whether political affiliations could be accurately judged from individuals' faces. To explore these judgments further, we asked participants to rate the faces of the targets from Study 2 along several trait dimensions previously found important for judgments of faces [7], [19]. We hypothesized that these trait ratings would provide clues to the differences between perceptions of Democrats' and Republicans' faces and explain the qualities that perceivers used to judge whether a target was Democrat or Republican.

Forty-six undergraduates (*n* = 30 women) participated in exchange for monetary compensation. Participants were instructed that they would be presented with a series of faces and that their task was to rate each face along several traits. The faces from college-aged Democrats and Republicans from Study 2 were presented in random order within randomly ordered blocks corresponding to each trait judgment. There were four blocks/traits in total: dominance, facial maturity, likeability, and trustworthiness. Each judgment was made along a 7-point scale anchored at each of *submissive*/*dominant* (Cronbach's α = .93), *babyish*/*mature* (Cronbach's α = .95), *not all likeable*/*very likeable* (Cronbach's α = .92), and *not at all trustworthy*/*very trustworthy* (Cronbach's α = .92). No participants recognized any of the targets, nor did they express any knowledge or suspicion that the faces varied in terms of political affiliation.

## Results

### Study 1

Data were analyzed using signal detection in which Democrat candidates categorized as Democrats were counted as hits (*M* = .52, *SD* = .07) and Republican candidates categorized as Democrats were counted as false-alarms (*M* = .44, *SD* = .09). Categorization accuracy and response bias were calculated using *A*' and *B*', respectively [22]. Participants were able to categorize targets according to their political affiliation significantly better than chance guessing [*M* = .57, *SD* = .08; *t*(28) = 4.41, *p*<.001, *r* = .64] and measures of response bias indicated that participants showed a slight tendency to categorize targets as Republicans more often than Democrats (*M* = .01, *SD* = .04).

These results did not change when participants indicating recognition of targets were excluded [*M*_A'_ = .57, *SD* = .09, *t*(18) = 3.39, *p* = .002, *r* = .54; *M*_B'_ = .01, *SD* = .02] and the accuracy [*t*(27) = 0.16, *p* = .87] and response bias scores [*t*(27) = 0.63, *p* = .53] of those participants recognizing targets versus those that did not recognize targets did not significantly differ. Similarly, Democrat and Republican participants did not differ in their accuracy [*t*(27) = 0.75, *p* = .46] or response bias [*t*(27) = 0.80, *p* = .43], nor did male versus female participants: *t*_A'_(27) = 1.56, *p* = .13; *t*_B'_(27) = 0.66, *p* = .52.

**Figure 1:**
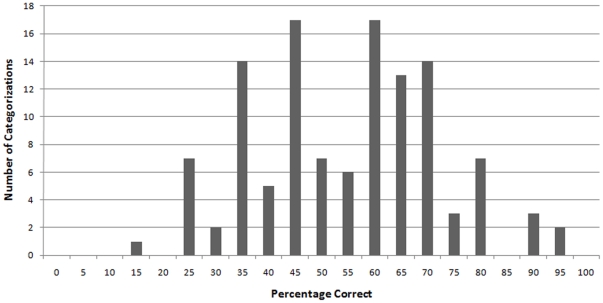
Frequency of accurate categorizations of targets as Democrats and Republicans in Study 1 according to 5% bins.

Additionally, to determine whether male and female politicians might be categorized differently, we calculated the percentage of correct categorizations for each target by aggregating across participants' categorizations. Comparison of the percentage of correct categorizations for male and female candidates showed no significant differences: *t*(116) = 1.48, *p* = .14. In addition, to be certain that the rates of accuracy observed here were not due to a small subset of easily categorized faces (e.g., targets who were obviously Democrats or Republicans), we plotted the percentage of correct categorizations for each target as a histogram in Figure 1 within 5% bins. This distribution shows that no face was categorized with complete accuracy (100%) or inaccuracy (0%). Rather, full consensus was not reached for any individual target and the categorizations were normally distributed (Shapiro-Wilk's *W* = .99, *p* = .37). Thus, it does not appear that a small number of faces is responsible for participants' overall accuracy. This suggests that these data do not speak to the ability to judge any single target as Democrat or Republican but, instead, speak to the general ability of the average perceiver to accurately discern others' political affiliations from their faces.

In sum, undergraduate perceivers were able to accurately categorize professional politicians according to their political affiliations. Judgments were not related to factors such as perceiver gender, politician gender, or the participants' own political affiliations. These data are consistent with previous research which has shown the importance of the face in expressing and interpreting social identities. As such, these data contribute additional evidence to the resilience of the perception and categorization of perceptually ambiguous groups.

The current study employed targets who were professional politicians. Moreover, these photos consisted of campaign photographs, which were undoubtedly carefully selected by the candidates and their staff. A question that remains from these findings, then, is whether the results are restricted only to targets for whom political affiliations are a core, public element of their identities. Might these effects generalize to other Democrats and Republicans whose political affiliations are not related to their careers? Study 2 explored this question by asking perceivers to categorize as Democrat and Republican the faces of college students belonging to Democrat and Republican clubs at their university.

### Study 2

**Figure 2:**
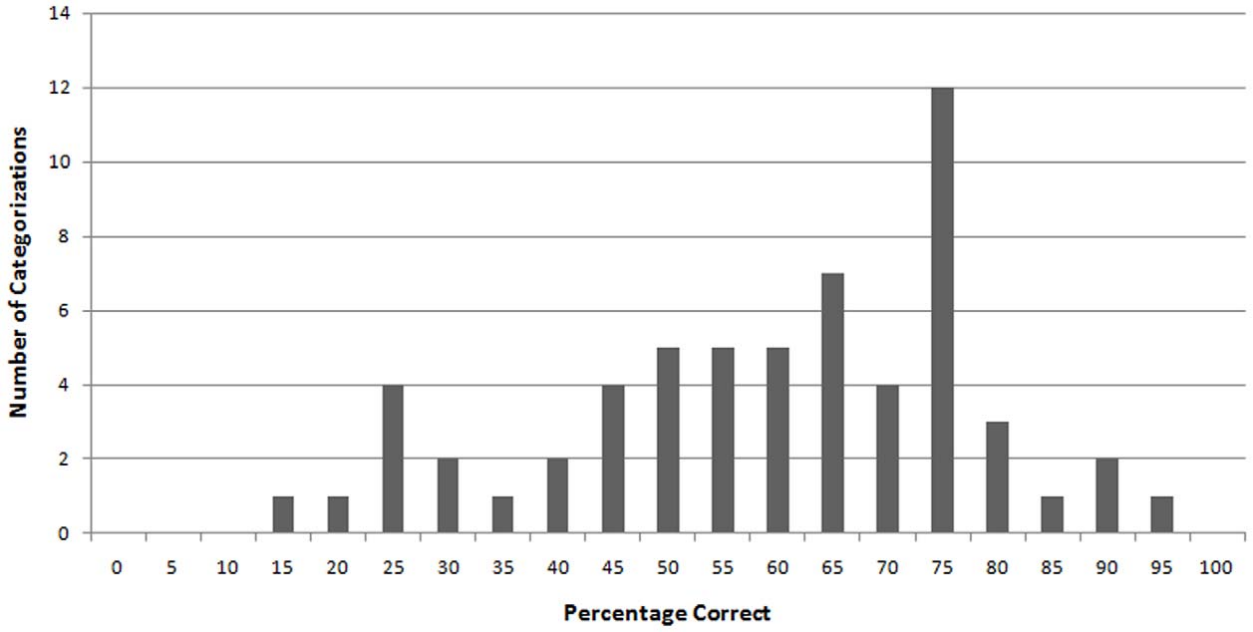
Frequency of accurate categorizations of targets as Democrats and Republicans in Study 2 according to 5% bins.

Data were analyzed using signal detection, as in Study 1 (Hits: *M* = .62, *SD* = .12; False-alarms: *M* = .48, *SD* = .08). Participants' categorizations of the targets' political affiliations were significantly greater than chance guessing [*M* = .62, *SD* = .12; *t*(23) = 4.91, *p*<.001, *r* = .72] and measures of response bias showed a proclivity among participants to categorize targets as Democrats more often than Republicans (*M* = −.05, *SD* = .11). Male and female participants did not significantly differ in either accuracy [*t*(23) = 1.67, *p* = .11] or response bias [*t*(23) = 0.93, *p* = .36]. Similarly, male and female targets did not significantly differ in the rates with which they were categorized according to their political affiliation: *t*(58) = 1.35, *p* = .18. Figure 2 shows the distribution of correct categorizations for each target within 5% bins. These frequencies show that no target was categorized with complete accuracy or inaccuracy and that perceivers did not reach complete consensus for any single face. Rather, the distribution of accuracy for the categorizations was normally distributed (Shapiro-Wilk's *W* = .96, *p* = .07), suggesting that participants' accuracy was based upon a general ability to infer political affiliation from targets' faces, not just the legibility of a small set of faces.

Congruent with the findings of Study 1, Study 2 therefore showed that political affiliations could be accurately discerned from the faces of college students, as displayed in their senior portraits. These data extend the finding that political affiliation can be gleaned from the face by showing that this effect is not restricted to professional politicians for whom their political group membership is a central element of their careers. In addition, the use of yearbook portraits, which are highly standardized and permit little opportunity for target-selection biases, provides a more controlled stimulus set than that of campaign photographs of political candidates.

What might be the basis for these judgments? To explore this, we asked a separate group of participants to provide ratings of the college students' faces along several relevant personality traits in Study 3.

### Study 3

The data for each trait were aggregated across participants to form a mean score for each target along each trait (descriptive statistics are presented in Table 1).We conducted a principal components factor analysis with varimax rotation, which suggested that the judgments constituted two factors. Factor 1 (46% of variance explained) consisted of high loadings on likeability (.94) and trustworthiness (.97) and low loadings on dominance (−.11) and facial maturity (.14). Factor 2 (42% of variance explained) consisted of high loadings on dominance (.92) and facial maturity (.89) and low loadings on likeability (.14) and trustworthiness (−.11). Consistent with previous studies examining judgments of faces along these traits [7], [19], we therefore formed a composite called Warmth by averaging together the targets' mean scores for likeability and trustworthiness and a composite called Power by averaging together the targets' mean scores for dominance and facial maturity.

**Table 1:** Descriptive statistics for the trait ratings in Study 3.

Trait	Democrat Targets		Republican Targets	
	Mean	Standard Deviation	Mean	Standard Deviation
Power	4.20	0.62	4.56	0.57
Dominance	3.93	0.74	4.30	0.62
Facial Maturity	4.46	0.63	4.82	0.66
Warmth	4.35	1.93	4.18	0.75
Likeability	4.31	0.69	4.18	0.86
Trustworthiness	4.40	0.62	4.18	0.68

We were first interested in whether Democrats and Republicans would differ in how they were judged according to Power and Warmth. We therefore correlated the mean scores for Power and Warmth with a dichotomous vector in which Republican targets were labeled as 0 and Democrat targets were labeled as 1. This analysis showed that Power scores were significantly related to targets' political affiliations [*r*(58) = −.30, *p* = .02] such that Republicans were perceived as significantly more powerful than were Democrats. There was no relationship between political affiliation and warmth: *r*(58) = .13, *p* = .33. Targets' gender may have affected these judgments, as women are typically seen as less powerful than men and there were more female Democrats than female Republicans [23]. To control for the influence of gender, we repeated the previous correlations but included a dichotomous vector coding male targets as 0 and female targets as 1 and included this as a covariate in the analysis. Statistically removing the effect of gender did not alter the results. That is, targets' political affiliations were still correlated with Power [*r*(57) = −.30, *p* = .02] but not with Warmth [*r*(57) = .13, *p* = .33].

**Figure 3:**
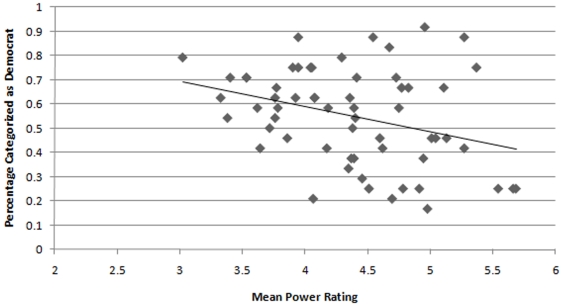
Relationship between the likelihood with which a target was perceived to be a Democrat in Study 2 and the mean Power rating for that target based on naïve judges' perceptions in Study 3.

**Figure 4:**
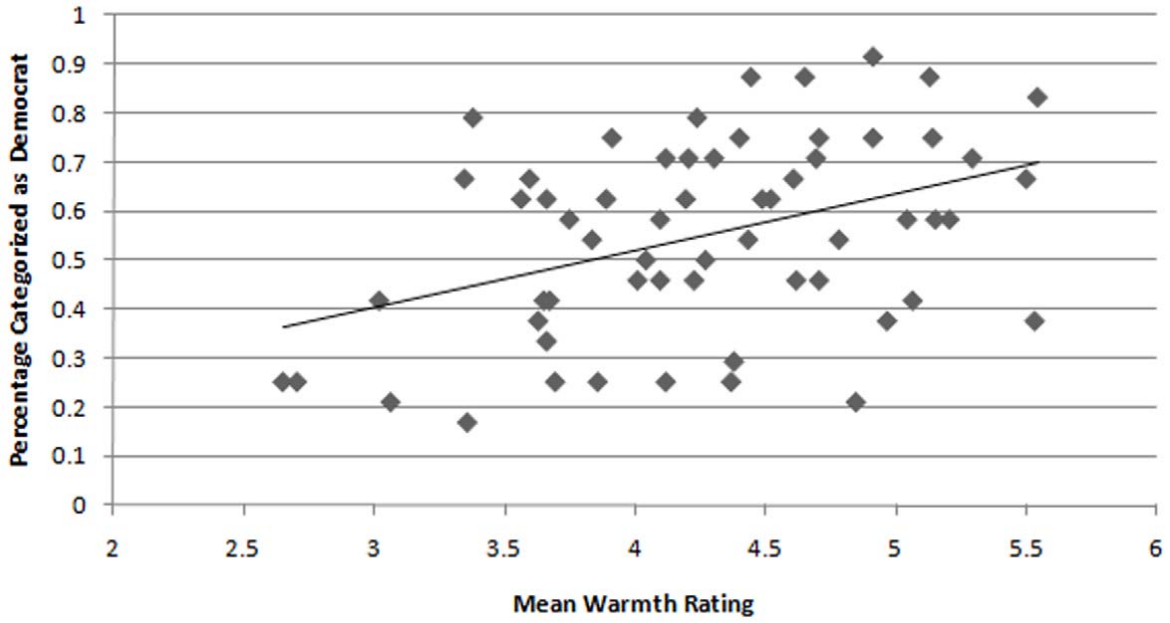
Relationship between the likelihood with which a target was perceived to be a Democrat in Study 2 and the mean Warmth rating for that target based on naïve judges' perceptions in Study 3.

We next wanted to explore how participants' perceptions of who were Democrats and Republicans (based on the judgments made in Study 2) might relate to perceptions of the targets in terms of Power and Warmth. We therefore calculated for each target the percentage of perceivers who had categorized the target as a Democrat in Study 2 and correlated these values with both Power and Warmth. This analysis showed that those targets perceived as Republicans were rated as greater in Power [*r*(58) = −.33, *p* = .01] whereas those targets perceived as Democrats were rated as greater in Warmth [*r*(58) = .40, *p* = .001]; see Figures 3 and 4. Again, statistically removing the effect of target gender did not change these results: *r*_Power_(57) = −.34, *p*<.01; *r*_Warmth_(57) = .39, *p* = .002.

**Figure 5:**
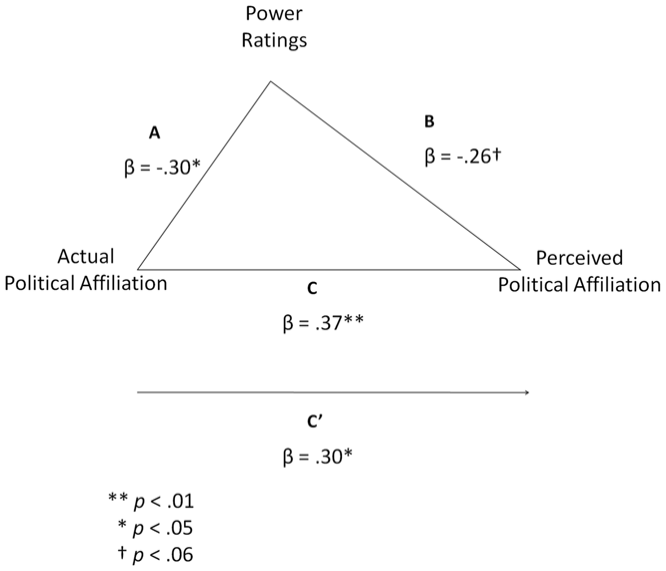
Mediation model with standardized regression coefficients showing the influence of participants' perceptions of Power as a partial mediator of the relationship between targets' actual political affiliations (coded as Republican  = 0, Democrat  = 1) and perceived political affiliations (coded as the percentage of participants who rated each target as a Democrat).

To better ascertain the influence of perceptions of Power on the categorizations of targets as Democrats and Republicans, we conducted a mediation analysis testing whether Power may mediate the relationship between perceptions of targets' political affiliations and their actual political affiliations. As illustrated in Figure 5, perceptions of Power partially mediated the relationship between targets' actual and perceived political affiliations: *Z* = 1.64, one-tailed *p* = .05.

Thus, Democrats and Republicans were perceived to possess different personality traits based on the appearance of their faces. Participants who were naïve to the differences in the targets' political affiliations rated the Republicans' faces as appearing more powerful than the Democrats' faces. More important, the perceptions of who were Democrats and Republicans in Study 2 were significantly related to particular traits. The faces of targets believed by more perceivers to be Republicans were seen as more powerful whereas the faces of targets believed by more perceivers to be Democrats were seen as more warm. The uneven distribution of men and women across the two parties in the sample did not affect these results. Moreover, perceptions of Power from the targets' faces partially mediated the relationship between the targets' actual and perceived political affiliations, suggesting a mechanism responsible for perceivers' judgments. These data therefore suggest that participants' categorizations of targets as Democrats and Republicans may relate to stereotypes of Democrats as warm and Republicans as powerful [24], [25].

## Discussion

People are adept at accurately inferring numerous traits and qualities of others based on their nonverbal behaviors and appearance. Consistent with this, here we found that both professional politicians' and college seniors' political affiliations could be accurately judged from static, grayscale photos of their faces. The basis for these effects appears to rest in perceivers' stereotypes of Republicans as appearing powerful and Democrats as appearing warm [24], [25], with perceptions of Power being a significant predictor of the targets' actual political affiliations.

These data extend what is known about our capacity to make reliable and accurate inferences of others based on their appearance. In particular, these findings add to the literature on the categorization of group memberships that are not perceptually obvious, such as sexual orientation [11] and religious group membership [14], [19]. Similar to what has been found for these other perceptually ambiguous groups, the effects for accuracy in Studies 1 and 2 were not driven by a subset of highly identifiable faces (as shown in Figures 1 and 2). Rather, the distribution of accuracy across targets—with none being entirely accurately categorized and none being entirely inaccurately categorized—represents a general and imperfect ability to accurately infer political group membership from nonverbal and appearance cues. This imperfection is similar to what is found for the distribution of accuracy in other perceptually ambiguous groups [12].

Moreover, the ability to judge political group membership from faces is explicated by perceivers' reliance on stereotypes to make their decisions. Study 3 showed that faces perceived as warm were likely to be those categorized as Democrats in Study 2 and that faces perceived as powerful were likely to be those categorized as Republicans in Study 2. Not surprisingly, these stereotypes lead to perceptual errors. Not at all Democrats appear warm and not all Republicans appear powerful. However, the linearity of these effects is noteworthy: appearing warmer led to a greater chance that a target would be perceived as a Democrat and appearing more powerful led to a greater chance that a target would be perceived as a Republican. Establishing the role of these perceptions in the categorizations provides a potential mechanism by which to explain the basis for perceivers' categorizations and perceptions of Power were found to partially mediate the relationship between targets' actual and perceived political affiliations. Moreover, Power was significantly related to the targets' actual political affiliations, whereas Warmth was not. Thus, perceptions of Power actually differentiated Republican and Democrat targets.

This connection between trait perceptions and stereotypes of Democrats and Republicans is not uncommon among the lay public [21], [24], [25]. Yet these stereotypes may differ depending on the perceiver's personal beliefs or environment. Indeed, one limitation of the current work is the paucity of Republican perceivers. Moreover, the studies were conducted in the northeastern U.S. where stereotypes about the personalities related to particular political affiliations may be different from those in other areas of the country. For instance, the northeastern U.S. is typically characterized by Democrat political leadership and support for liberal legislative perspectives [26]. The finding that outgroup Republicans are seen as powerful (i.e., dominant and facially mature) and ingroup Democrats are seen as warm (i.e., likeable and trustworthy) could be confounded by the perceivers' expectations, beliefs, and desires for Democrat and Republican personalities. Although the perceivers in Study 3 did not know that they were rating targets who differed systematically on political group membership, further testing of these effects with a larger percentage of Republican perceivers would be useful for better ascertaining the nature of these effects.

In sum, the finding that political affiliation can be accurately judged from targets' faces extends our knowledge of the power of facial cues in forming accurate impressions of others. In addition, the grounding of these effects in perceivers' naïve inferences of the personality traits of the targets provides a potential mechanism for understanding the basis of these effects.

## References

[1] Ambady N, Bernieri FJ, Richeson JA (2000). Toward a histology of social behavior: Judgmental accuracy from thin slices of the behavioral stream.. Adv Exp Soc Psychol.

[2] Zebrowitz LA (1997). Reading faces: Window to the soul?.

[3] Macrae CN, Bodenhausen GV (2000). Social cognition: Thinking categorically about others.. Ann Rev Psychol.

[4] Collins MA, Zebrowitz LA (1995). The contributions of appearance to occupational outcomes in civilian and military settings.. J Appl Soc Psychol 25:.

[5] Zebrowitz LA, McDonald SM (1991). The impact of litigants' baby-facedness and attractiveness on adjudications in small claims courts.. Law Hum Behav.

[6] Livingston RW, Pearce NA (2009). The teddy-bear effect: Does having a baby face benefit black chief executive officers?. Psychol Sci.

[7] Rule NO, Ambady N (2008). The face of success: Inferences from Chief Executive Officers' appearance predict company profits.. Psychol Sci.

[8] Rule NO, Ambady N (2009). She's got the look: Inferences from female chief executive officers' faces predict their success.. Sex Roles.

[9] Brown E, Perrett DI (1993). What gives a face its gender?. Perception.

[10] Roberts T, Bruce V (1988). Feature saliency in judging the sex and familiarity of faces.. Perception.

[11] Rule NO, Ambady N, Adams RB, Macrae CN (2008). Accuracy and awareness in the perception and categorization of male sexual orientation.. J Pers Soc Psychol.

[12] Rule NO, Ambady N, Hallett KC (2009). Female sexual orientation is perceived accurately, rapidly, and automatically from the face and its features.. J Exp Soc Psychol.

[13] Rule NO, Macrae CN, Ambady N (2009). Ambiguous group membership is extracted automatically from faces.. Psychol Sci.

[14] Rice DR, Mullen B (2003). Isaac, Ishmael, and Janus: Past and future lessons regarding the ethnic categorization of faces.. App Cogn Psychol.

[15] Rule NO, Ambady N (2008). Brief exposures: Male sexual orientation is accurately perceived at 50 ms.. J Exp Soc Psychol.

[16] Rule NO, Garrett JV, Ambady N (2010). Faces and places: Geographic environment influences the ingroup memory advantage.. J Pers Soc Psychol.

[17] Ballew CC, Todorov A (2007). Predicting political elections from rapid and unreflective face judgments.. Proc Natl Acad Sci U S A.

[18] Poutvaara P, Jordahl H, Berggren N (2009). Faces of politicians: Babyfacedness predicts inferred competence but not electoral success.. J Exp Soc Psychol.

[19] Rule NO, Ambady N, Adams RB, Ozono H, Nakashima S (2010). Polling the face: Prediction and consensus across cultures.. J Pers Soc Psychol.

[20] Antonakis J, Dalgas O (2009). Predicting elections: Child's play!. Science.

[21] Elms AC (1976). Personality in politics..

[22] Quanty MB, Keats JA, Harkins SG (1975). Prejudice and criteria for identification of ethnic photographs.. J Pers Soc Psychol.

[23] Chiao JY, Bowman NE, Gill H (2008). The political gender gap: Gender bias in facial inferences that predict voting behavior.. PLoS ONE.

[24] Hayes D (2005). Candidate qualities through a partisan lens: A theory of trait ownership.. Amer J Poli Sci.

[25] Hayes D (2009). Feminine Democrats, masculine Republicans: Gender and party stereotyping in candidate trait attributions.. http://faculty.maxwell.syr.edu/dwhayes/femdems_may09.pdf.

[26] Gelman A (2008). Red state, blue state, rich state, poor state..

